# Structure and function of a novel GH8 endoglucanase from the bacterial cellulose synthase complex of *Raoultella ornithinolytica*

**DOI:** 10.1371/journal.pone.0176550

**Published:** 2017-04-27

**Authors:** Sandra Mara Naressi Scapin, Flavio Henrique Moreira Souza, Leticia Maria Zanphorlin, Thamyres Silva de Almeida, Youssef Bacila Sade, Alexander Machado Cardoso, Guilherme Luiz Pinheiro, Mario Tyago Murakami

**Affiliations:** 1 Division of Metrology Applied to Life Sciences, National Institute of Metrology, Quality and Technology, Duque de Caxias, Rio de Janeiro, Brazil; 2 Brazilian Bioethanol Science and Technology Laboratory, National Center for Research in Energy and Materials, Campinas, São Paulo, Brazil; Weizmann Institute of Science, ISRAEL

## Abstract

Cellulose synthesis in bacteria is a complex process involving the concerted action of several enzymes whose genes are often organized in operons. This process influences many fundamental physiological aspects such as bacteria and host interaction, biofilm formation, among others. Although it might sound contradictory, the participation of cellulose-degrading enzymes is critical to this process. The presence of endoglucanases from family 8 of glycosyl hydrolases (GH8) in bacterial cellulose synthase (Bcs) complex has been described in different bacteria, including the model organism *Komagataeibacter xylinus*; however, their role in this process is not completely understood. In this study, we describe the biochemical characterization and three-dimensional structure of a novel GH8 member from *Raoultella ornithinolytica*, named AfmE1, which was previously identified by our group from the metagenomic analysis of the giant snail *Achatina fulica*. Our results demonstrated that AfmE1 is an endo-β-1,4-glucanase, with maximum activity in acidic to neutral pH over a wide temperature range. This enzyme cleaves cello-oligosaccharides with a degree of polymerization ≥ 5 and presents six glucosyl-binding subsites. The structural comparison of AfmE1 with other GH8 endoglucanases showed significant structural dissimilarities in the catalytic cleft, particularly in the subsite +3, which correlate with different functional mechanisms, such as the recognition of substrate molecules having different arrangements and crystallinities. Together, these findings provide new insights into molecular and structural features of evolutionarily conserved endoglucanases from the bacterial cellulose biosynthetic machinery.

## Introduction

Bacterial Cellulose Synthase (Bcs) complexes are present in cellulose-producing bacteria and comprise a variety of subunits that act on the concerted export and arrangement of the growing polysaccharide chain on the cell surface. In these bacteria, cellulose and its derivatives constitute critical components of the extracellular matrix and play key roles in biofilm formation and cell-cell interactions, therefore contributing to the modulation of virulence of important plant and human pathogens. The genes coding for Bcs subunits are typically arranged in operons and a growing diversity of them has being revealed by genomic data analysis (reviewed in [1]). Surprisingly, the ubiquitous presence of cellulase-coding genes in these operons has been described, including endoglucanase and β-glucosidase genes (referred in general as bcsZ and bglX, respectively).

Amongst the bcsZ genes, sequences coding for endoglucanases of the glycosyl hydrolase family 8 (GH8) have been found in Bcs operons. Growing evidence has demonstrated the importance of these enzymes in the synthesis of bacterial cellulose, especially to the correct packing of cellulose microfibrils, though their exact role in this process is still unknown. Most evidences came from studies with the protein CMCax from *Komagataeibacter xylinus*, a model organism for studying cellulose biosynthesis. Overproduction of CMCax in *K*. *xylinus* or even the addition of an endoglucanase to the bacterial culture increased cellulose synthesis [2, 3]. Furthermore, antibodies against CMCax inhibited the production of cellulose fibrils [4] and gene disruption remarkably reduced the cellulose yield probably due to the formation of defective and highly twisted fibrils [5].

Studies with the protein CelC2 from the nitrogen-fixing bacterium *Rhizobium leguminosarum* have also shown the critical role of a GH8 endoglucanase in cellulose biosynthesis with important impacts in the primary and secondary symbiotic infection of host roots [6, 7], as well as in the formation of active biofilms on plant roots and abiotic surfaces [8]. Similarly, GH8 endoglucanases required for cellulose synthesis were described in *Agrobacterium tumefaciens* [9] and *Escherichia coli* [10].

Furthermore, endoglucanases are also essential components of the cellulose synthase complex in plants, where they originated from cyanobacteria (chloroplast ancestors) [11]. These enzymes, termed Korrigan (KOR), were firstly isolated in an *Arabidopsis thaliana* dwarf mutant and belong to family GH9, which share a remote fold similarity with GH8 endoglucanases [12, 13].

Structural and functional characterization of different components of cellulose synthase complexes both in bacteria and plants provides an important basis to fully understand the process of cellulose production and its diversity [1, 14], as well as of symbiotic or pathogenic infection and biofilm formation [15]. Moreover, it may also impact on biotechnological applications such as the production of new biopolymers [16].

In the present study, we describe a comprehensive biochemical, biophysical and structural characterization of a novel family 8 endoglucanase from *Raoultella ornithinolytica*, named AfmE1 (***A****chatina* ***f****ulica* **m**etagenome **E**ndoglucanase **1**) since its sequence was identified in the metagenome of the giant African snail *A*. *fulica* [17]. Our results demonstrate the key enzymatic properties of AfmE1 including its specificity, mode of action and kinetic parameters. In addition, high-resolution crystallographic analysis revealed structural differences in the substrate-binding cleft, which is related to the recognition and processing of distinct substrates. Altogether, these results shed light on the mechanistic basis of the major endoglucanase factor of the Bcs complex from the bacterium *Raoultella ornithinolytica*, a lignin-degrading bacterium from forest soil.

## Materials and methods

### Molecular cloning, expression and purification

Plasmid pUC-AfmE1, containing AfmE1 coding sequence optimized for expression in *E*. *coli* and lacking its signal peptide, was synthesized by the company Genscript (Piscataway, NJ, USA). This sequence was subcloned into the restriction sites NdeI and XhoI of the pET28a expression vector. Recombinant gene expression was carried out in *E*. *coli* Rosetta™(DE3) transformed with plasmid pET28a-AfmE1 and grown in LB medium containing the antibiotics kanamycin and chloramphenicol. The cells were induced with 0.5 mM IPTG (isopropyl β-D-thiogalactopyranoside) at 20°C for 16 h, then harvested and resuspended in buffer containing 50 mM Tris-HCl pH 8.0, 100 mM NaCl, 5% (v/v) glycerol and 1 mM PMSF (phenylmethylsulfonyl fluoride). Cells were lysed with lysozyme (100 μg/mL) on ice for 1 h, followed by sonication in a Q700 sonicator (QSonica, Newtown, CT, USA) with 60 pulses of 5 s at 30 W. The extracts were clarified by centrifugation (20,000 g, 20 min and 4°C) and loaded at a flow-rate of 1 mL/min onto a 5 mL His-Trap FF column equilibrated with 20 mM Tris-HCl pH 8.0 containing 20 mM NaCl and coupled to an ÄKTA FPLC system (GE Healthcare Biosciences, Pittsburgh, PA, USA). Proteins were eluted using a 75 mL linear gradient from 0 to 500 mM imidazole. The eluted fractions were analyzed by SDS-PAGE [18] and those containing purified AfmE1 were pooled and dialyzed against 20 mM Tris-HCl buffer at pH 7.4. Protein concentration was estimated from direct absorbance at 280 nm using Nanodrop (Thermo Scientific, Waltham, MA, USA), considering the molar extinction coefficient and the theoretical protein molecular mass deduced from the 6xHis-AfmE1 coding sequence. As the N-terminal 6xHis-tag did not interfere with protein activity, it was maintained in all the subsequent experiments.

### Enzyme assays

The optimal reaction conditions and kinetic parameters for AfmE1 were established using the 3,5-dinitrosalicylic acid (DNS) method to measure the reducing sugars released as an indicator of enzyme hydrolytic activity [19]. Substrate specificity assays were performed at 40°C in reactions containing 30 μg.mL^-1^ enzyme in 50 mM sodium phosphate buffer at pH 6.5 and 1–2% (w/v) of different polysaccharide substrates: carboxymethyl cellulose 4M (CMC 4M), low viscosity carboxymethyl cellulose, lichenan (Icelandic Moss), arabinogalactan (Larch Wood), pullulan, xyloglucan (Tamarind), glucomannan (Konjac), arabinan (Sugar Beet), β-glucan (Barley, low viscosity), arabinoxylan (Wheat), mannan (Ivory Nut) and curdlan. All the substrates were purchased from Megazyme (Wicklow, Ireland), except for low viscosity CMC that was purchased from Sigma-Aldrich Co., St. Louis, MO, USA (CMC, low viscosity). The pH activity dependence was determined using McIlvaine’s buffer (pH range 3.5–8.0) containing 1% (w/v) of substrate (CMC or β-ᴅ-glucan) at 40°C for 10 min. To determine the optimal temperature range, the reactions were performed in 10 mM citrate buffer at pH 6.0 containing 1% (w/v) CMC at temperatures from 20 to 70°C. The thermostability analysis was performed by incubating the enzyme at temperatures from 35 to 60°C during different times and measuring its residual activity under optimal reaction conditions afterwards. Kinetic parameters (*K*_m_, *V*_max_ and *k*_cat_) were determined by measuring the initial rate of reaction at β-glucan concentrations ranging from 0.7 to 40 mg/L for 10 min under optimal reaction conditions. The assays were performed in quadruplicate and the data were fitted to the Michaelis-Menten equation. One unit (U) was defined as the amount of enzyme required to release 1 μmol of reducing sugar per minute.

### Capillary zone electrophoresis and thin-layer chromatography

The products generated by the hydrolytic activity of AfmE1 against β-ᴅ-glucan and CMC were analyzed by capillary zone electrophoresis (CZE). Enzymatic hydrolysis of cello-oligosaccharides was analyzed by thin-layer chromatography (TLC) and capillary zone electrophoresis (CZE). For TLC analysis, 2 μL of hydrolyzed samples were spotted onto Silica gel-60 plates (DC-Fertigfolien Alugram Xtra SIL G from Macherey-Nagel GmbH and Co., Düren, Germany) and developed using as mobile phase a mixture of ethyl acetate, acetic acid and water in a volume ratio of 3:2:1, as previously described [20]. After 90 min of separation, the TLC plate was air-dried and then sprayed with a mixture of 0.3% (w/v) α-naphthol and 5% (v/v) sulfuric acid in methanol and heated to 100°C for 10 min to visualize the resolved products. Glucose (C1), cellobiose (C2), cellotriose (C3), cellotetraose (C4), cellopentaose (C5) and cellohexaose (C6) (Sigma-Aldrich Co., St. Louis, MO, USA) were used as standards. For CZE analysis, the reaction products were derivatized with 8-aminopyrene-1,3,6-trisulfonic acid (APTS) by reductive amination [21] and then analyzed in a P/ACE MD instrument configured with a laser-induced fluorescence detection system (Beckman Coulter, Brea, CA, USA). An uncoated fused silica capillary of 75 μm internal diameter and 20 cm effective length (Beckman Coulter) conditioned with 40 mM potassium phosphate (pH 2.5) was used for analysis of APTS-labeled sugars. The samples were injected by application of 0.5 p.s.i. for 5 s and the electrophoretic conditions were 20 kV/70–100 mA with reverse polarity at a controlled temperature of 25°C. Labeled oligosaccharides were excited at 488 nm, and emission was collected through a 520-nm bandpass filter. The resultant peaks were assigned by comparison with electrophoretic behavior of glucose and cello-oligosaccharide standards.

### Circular dichroism spectroscopy

Circular dichroism (CD) spectra were acquired on a JASCO J-815 CD spectrometer controlled by a CDF-426S/15 Peltier temperature control system (Jasco Analytical Instruments, Easton, MD, USA) using a quartz cuvette with a 1-cm path length. The enzyme was prepared in phosphate buffer (20 mM sodium phosphate, 150 mM NaCl, pH 7.5) at a final concentration of 8 μM. All spectra were obtained at 20°C in the range 200–260 nm with a bandwidth of 2 nm and a response time of 4 s/nm. CD spectra were buffer subtracted and normalized to mean residue ellipticity. Thermal unfolding experiments were monitored at 222 nm in the temperature range 20–90°C with a scan rate of 1°C.min^-1^. The melting temperature (*T*_M_) was determined according to the sigmoidal-Boltzmann fitting of the CD denaturation curve.

### Dynamic light scattering

In solution size distribution of the purified enzyme was evaluated using dynamic light scattering. Measurements were acquired at 20°C on a Malvern Zetasizer Nano ZS 90 Model No. 3690 (Malvern Instruments, Worcestershire, UK) with a 633 nm laser, in a quartz cell with a scattering angle of 90°. The protein was analyzed at a concentration of 0.5 mg.mL^-1^ in phosphate buffer (20 mM sodium phosphate, 150 mM NaCl, pH 7.5). An average of 20 runs was used to estimate the hydrodynamic radius (*R*_H_) through Stokes-Einstein equation.

### Analytical ultracentrifugation

Sedimentation velocity experiments were performed on a Beckman Optima XL-A analytical ultracentrifuge (Beckman Coulter Inc., Indianapolis, IN, USA) at 20°C. Data were collected at both 220 and 280 nm. The protein was prepared in different concentrations ranging from 0.2 to 0.9 mg.mL^-1^ in phosphate buffer (20 mM sodium phosphate, 150 mM NaCl, pH 7.5). AUC data were analyzed using the continuous sedimentation distribution method in the SEDFIT program. The *s*^0^_20,w_ value at infinite dilution was calculated by linear regression of *s*_20,w_ as a function of protein concentration.

### Differential scanning calorimetry

Thermal stability was also analyzed by differential scanning calorimetry (DSC) using a VP-DSC device (Microcal, Malvern Instruments Inc., Westborough, MA, USA). The enzyme was prepared in phosphate buffer (20 mM sodium phosphate, 150 mM NaCl and pH 7.5) at a final concentration of 2 mg.mL^-1^. A temperature rate of 1°C.min^-1^ was used and the reversibility of protein denaturation was tested. Denaturation curves were buffer subtracted, concentration normalized and the resultant endotherms integrated following assignment of pre- and post-transition baselines.

### Protein crystallization

The purified AfmE1 dialyzed against 20 mM Tris-HCl, pH 7.4 was concentrated to 11.5 mg.mL^-1^ using Amicon Ultra centrifugal filter units (Millipore, Billerica, MA, USA). Crystallization experiments were performed by the vapor diffusion method using a HoneyBee 963 robot (Digilab, Marlborough, MA, USA). Sitting drops were prepared by mixing 1.0 μL of the protein solution with an equal volume of mother liquor and equilibrated against 80 μL of the reservoir solution at 18°C. A total of 544 formulations based on commercial crystallization kits were tested, including SaltRX, Crystal Screen, Crystal Screen 2 (Hampton Research, Aliso Viejo, CA, USA), Precipitant Synergy, Wizard I and II (Emerald BioSystems, Bainbridge Island, WA, USA), PACT and JCSG (Qiagen Canada Inc., Montreal, Canada). Crystals of AfmE1 were observed in different conditions and better diffraction quality was obtained from crystals grown in 7 days in the solution containing 10% (w/v) polyethylene glycol (PEG) 1000 and 10% (w/v) PEG 8000.

### X-ray diffraction data collection and processing

A single crystal (300x40x20 μm^3^) was soaked in a cryoprotectant solution consisting of the mother solution with 20% (v/v) glycerol and flash cooled in a nitrogen-gas stream at 100 K for data collection. Diffraction data were collected at the MX2 beamline (LNLS, Campinas, SP, Brazil) with the wavelength fixed at 1.458 Å. A fine-slicing strategy was used (0.2° per frame and 5 s of exposure time) and 180° were recorded using a Pilatus 2M detector (Dectris, Baden-Dättwil, CH). The crystal-to-detector distance was set to a maximum resolution of 2.2 Å. The data were indexed, integrated and scaled using the XDS package [22]. Data collection and processing statistics are summarized in Table 1.

**Table 1:** Data collection and refinement statistics.

Data collection and Processing	
Wavelength (Å)	1.458
Resolution range (Å)	44.61–2.39 (2.48–2.39)
Space group	P2_1_2_1_2_1_
Unit cell (Å)	a = 50.42, b = 76.37, c = 95.66
Total reflections	78,940 (5,903)
Unique reflections	14,895 (1,294)
Multiplicity	5.3 (4.6)
Completeness (%)	98.2 (87.7)
Mean I/sigma(I)	7.03 (1.26)
Wilson B-factor	44.9
R-merge	0.159 (1.292)
R-meas	0.176
CC_1/2_	0.99 (0.61)
Refinement	
*R*_work_	0.2011 (0.3215)
*R*_free_	0.2475 (0.3477)
Protein residues	315
Ligands	2 (PO_4_)
Solvent molecules	51
RMS(bonds) (Å)	0.009
RMS(angles) (°)	1.18
Ramachandran analysis (%)	
Favoured	95
Allowed	5
Outliers	0
Average B-factor (Å^2^)	48.00

### Structure determination and refinement

The AfmE1 crystal structure was solved by molecular replacement using the program Phaser [23] and the atomic coordinates of the endoglucanase CMCax from *Komagataeibacter xylinus* (PDB code 1WZZ, [24]). The crystallographic structure was refined by interspersed cycles of manual model building based on inspection of the 2Fo–Fc and Fo–Fc maps using the program COOT [25] and restrained and isotropic B-factor refinement with the program REFMAC5 [26]. Translation, libration, and screw-motion (TLS) parameters were applied in the final refinement cycles. The local and global model quality was assessed using MolProbity [27] and refinement statistics are shown in Table 1. The atomic coordinates and structure factors have been deposited in the RCSB Protein Data Bank (http://www.rcsb.org/) with the accession code 5CZL. Figures were generated using the program Pymol [28].

## Results and discussion

### AfmE1 is a putative endoglucanase from *Raoultella ornithinolytica* and colocalizes with several genes encoding Bcs complex proteins

AfmE1 (***A****chatina* ***f****ulica* **m**etagenome **E**ndoglucanase **1**) gene sequence was identified through analysis of ORFs (open reading frames) encoding endoglucanases present in the *A*. *fulica* metagenome (GenBank: SRA051264; [17]). Sequence alignment against the non-redundant NCBI database showed that the recovered sequence is 100% identical to WP_015585553.1, annotated as a coding sequence for a putative endoglucanase belonging to the glycosyl hydrolase family 8 (GH8) from *Raoultella ornithinolytica* strain S12 (NCBI genome accession: CP010557.1), a lignin-degrading bacterium from forest soil [29]. Furthermore, AfmE1 shares significant sequence identity to some known GH8 endoglucanases, including an enzyme from the *Cellulomonas uda* strain CB4 (Uniprot: P18336) and the cellulase CelY from *Dickeya dadantii* strain 3937 (previously named as *Erwinia chrysanthemi* (Uniprot P27032, [30]), supporting that AfmE1 constitutes a new GH8 family member. *C*. *uda* CB4 is a strain isolated from brewery sewage in Japan that presents a high-titer cellulase production [31]. The *C*. *uda* endoglucanase gene was expressed in the ethanologenic bacterium *Zymomonas mobilis* to increase its substrate range and produce high levels of activity [32]. *D*. *dadantii* CelY acts synergistically with cellulase CelZ in the hydrolysis of CMC and amorphous cellulose [33] and increases the hydrolysis rate of crystalline cellulose in the presence of low levels of commercial cellulases of fungal origin [34].

**Fig 1:**
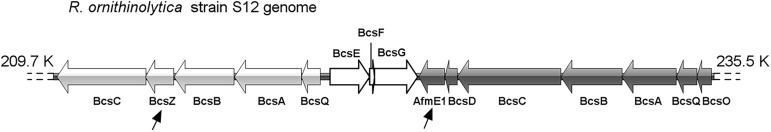
Schematic representation of genes coding for cellulose synthase subunits in a section of *R*. *ornithinolytica* strain S12 genome. Bcs genes are arranged in operons, as illustrated. The genes coding for the endoglucanases AfmE1 and BcsZ are indicated by arrows. According to the classification of Bcs operons proposed by Römling & Galpering [1], AfmE1 and BcsZ integrate distinct operons of the subtypes Ib and IIa, respectively. NCBI genome accession: CP010557.1.

Interestingly, AfmE1 also shares approximately 36% sequence identity with CMCax from *Komagataeibacter xylinus*, a cellulase known to be involved in the process of cellulose biosynthesis in this organism, and the analysis of the genes close to AfmE1 sequence in the genome of *R*. *ornithinolytica* demonstrated its colocalization with several genes encoding Bcs complex proteins (Fig 1). Furthermore, the gene for the aforementioned protein CelY from *D*. *dadantii* also colocalizes with a Bcs operon in the bacterial genome (GenBank accession number CP002038; [35]), although this enzyme has never been previously related to cellulose biosynthesis. These observations may associate AfmE1 protein with cellulose biosynthesis mechanisms, expanding its biotechnological potential beyond the breakdown of polysaccharides. To clarify this issue, functional and structural studies have been conducted to investigate AfmE1 role at the molecular level.

### AfmE1 cleaves β-1,4 glucopyranosyl linkages and presents high catalytic activity in a broad temperature range

The full-length AfmE1 was expressed in *E*. *coli* fused to a 6xHis-tag at the N-terminus and purified to homogeneity. As the GH8 family encompasses different classes of enzymes, with different specificities, AfmE1 hydrolytic activity was tested against various substrates, including CMC, lichenan, arabinogalactan, pullulan, xyloglucan, glucomannan, arabinan, β-glucan, arabinoxylan, mannan and curdlan. The results demonstrated that AfmE1 effectively cleaves β-glucan, CMC, and also, to a lesser extent, lichenan (data not shown). Therefore, AfmE1 seems to preferentially hydrolyze β-1,4 glucopyranosyl bonds.

**Fig 2:**
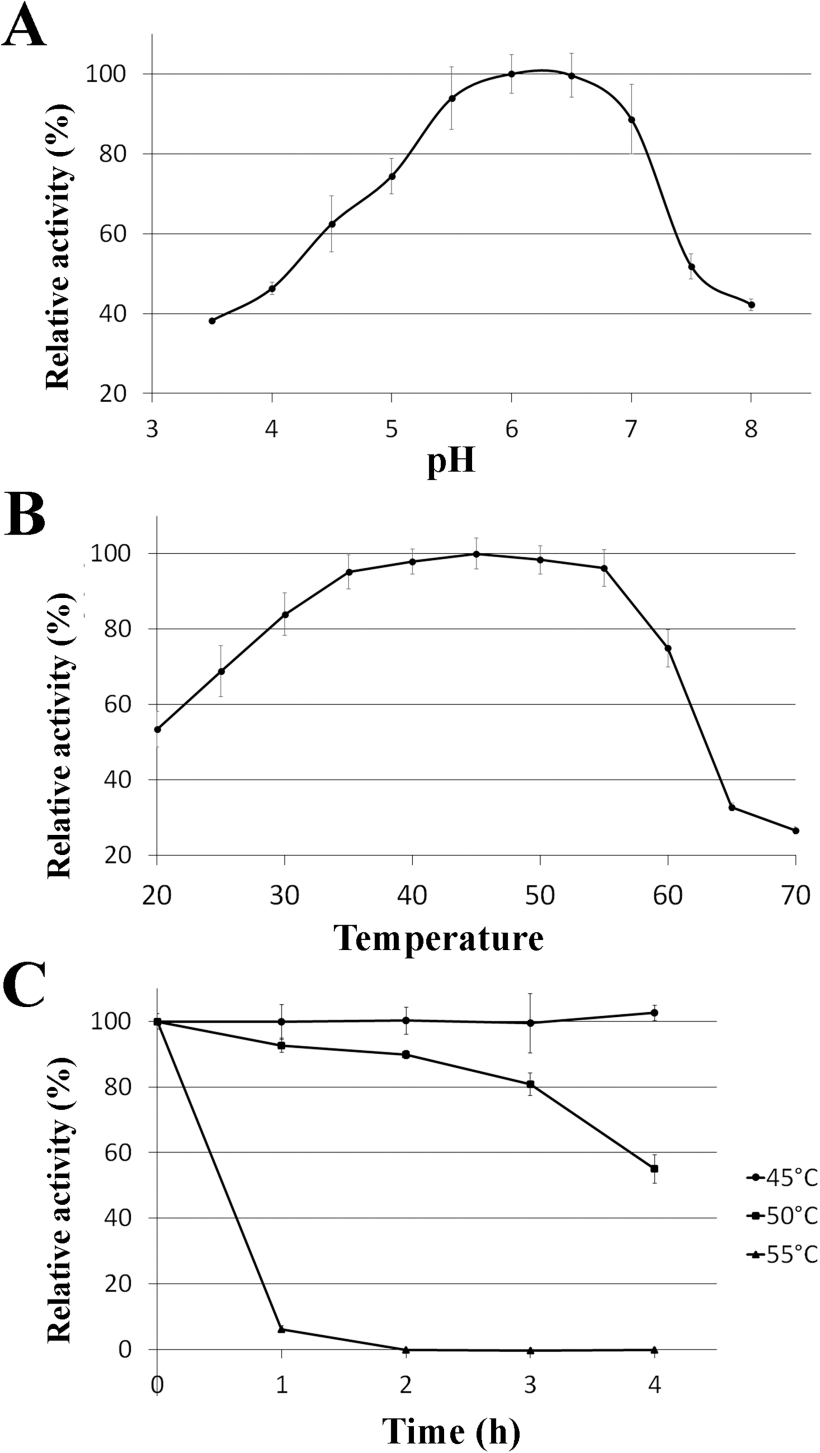
Effect of pH and temperature on the catalytic activity of AfmE1. (A) Determination of optimum pH. The hydrolytic activity was measured at different pHs at 40°C for 10 min. (B) Determination of optimum temperature. The hydrolytic activity was measured at temperatures ranging from 20 to 70°C. (C) Thermal stability assay. The enzyme was incubated at 45, 50 and 55°C for up to 4 h and residual activity was determined under the optimal reaction conditions. Error bars represent the standard deviation.

The effects of pH on the enzyme activity were measured (Fig 2A and Table 2). The optimum pH range (more than 80% relative activity) was between 5.5 and 7.0, with maximum activity at pH 6.0 to 6.5. The pH dependence curve shows that the enzyme is more sensitive to alkaline than acidic pH, as the activity decreases rapidly in pH values higher than 7.0. The enzymatic activity and stability of AfmE1 were also evaluated at different temperatures (Fig 2B and 2C and Table 2). The optimal temperature of the recombinant enzyme was 45°C, but high catalytic activity was noticed over a broad temperature range, from 30 to 55°C. The enzyme is stable at temperatures up to 45°C for at least 4 h and at 50°C for 3 h. Similar biochemical parameters were also observed for other GH8 enzymes such as those from *Komagataeibacter xylinus* [2], *Klebsiella pneumoniae* [36], *Clostridium cellulolyticum* [37], *Bacillus* sp. [38] and *Halomonas* sp. [39].

**Table 2:** Biochemical characterization of AfmE1.

Kinetic parameters (β-glucan)	
*K*_m_ (mg.mL^-1^)	8.6 + 0.47
*k*_cat_ (s^-1^)	47.4 + 2.62
*k*_cat_/*K*_m_ (mL.s^-1^.mg^-1^)	5.5 + 0.30
V_0_ at [S] = 10 mg/mL (U.mg^-1^)	
CMC 4M	43.3 + 2.44
β-glucan	40.4 + 3.43
Lichenan	23.9 + 3.20
pH and temperature parameters	
pH range (optimum)	5.5–7.0 (6.0–6.5)
Temperature range (optimum)	30–55°C (45°C)
Thermal stability	≥ 4 h at *T* ≤ 45°C, 3 h at *T* = 50°C
Melting temperature (*T*_M_)	55°C

Kinetic studies were performed under optimal pH and temperature. The Michaelis-Menten parameters toward β-glucan were determined from the saturation curve obtained using different substrate concentrations (Table 2, S1 Fig). The values for *K*_m_, *V*_max_ and the catalytic constant *k*_cat_ were 8.6 mg.mL^-1^, 74.9 U.mg^-1^ and 47.4 s^-1^, respectively. Family GH8 endoglucanases from *Clostridium thermocellum* [40], *Bacillus circulans* [41], *Halomonas* sp. [39] and Ladakh soil metagenome [42] showed catalytic efficiencies of 1.5, 3.7, 2.1 and 2.5 mL.mg^-1^.s^-1^, respectively. Based on our data, the catalytic efficiency (*k*_cat_/*K*_m_) of AfmE1 was 5.5 mL.mg^-1^.s^-1^, which is higher than what has been described for other GH8 endoglucanases so far.

In the kinetic analyses using CMC or lichenan as substrate, saturation was not achieved. Nevertheless, for comparison purposes, initial reaction rates (*v*_0_) were determined for CMC, β-glucan and lichenan at the same substrate concentration (10 mg/mL) (Table 2). The reaction rates with CMC and β-glucan are quite similar and almost twice as fast as that in the presence of lichenan (S1 Fig). This fact can be attributed to the higher proportion of β-1,3 glycosidic bonds in lichenan, relative to the β-1,4 linkages that are preferentially hydrolyzed by AfmE1.

### AfmE1 is an endo-acting enzyme and hydrolyzes cello-oligosaccharides containing at least five glucose units

**Fig 3:**
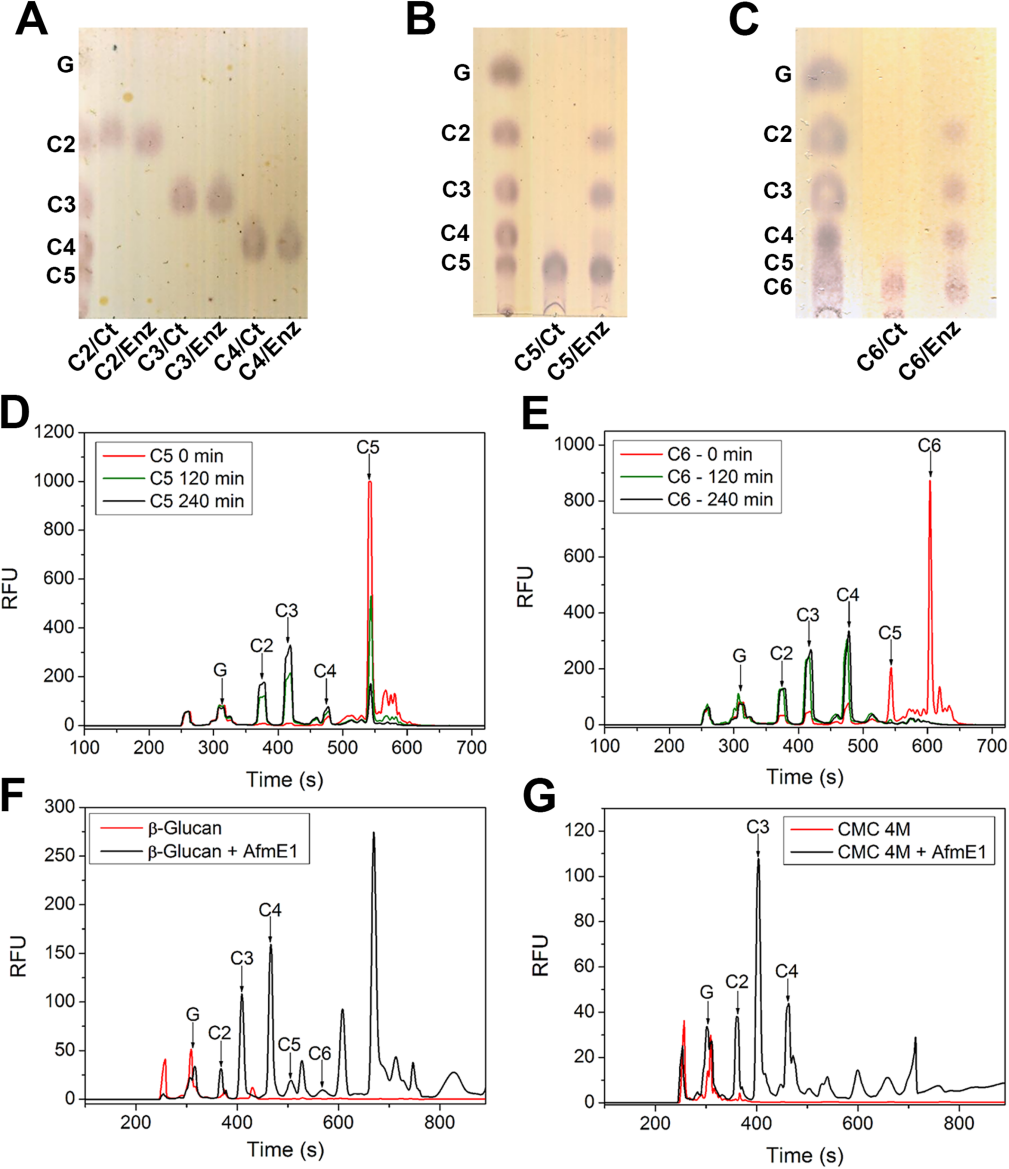
AfmE1 mode of action. Thin-layer chromatography analysis of degradation products derived from AfmE1-mediated hydrolysis of different cello-oligosaccharides. (A) cellobiose, cellotriose and cellotetraose; (B) cellopentaose and (C) cellohexaose. The first line of each panel corresponds to a mixture of the indicated standards. CZE electropherograms of the APTS-labeled products of cellopentaose (D) and cellohexaose (E) hydrolysis after 0, 2 and 4 h of incubation with AfmE1. CZE electropherograms of the APTS-labeled products from AfmE1-mediated hydrolysis of β-glucan (F) and CMC (G). The labeled cello-oligosaccharides are indicated, as inferred from a parallel run of a standard mixture. For all the analyses, control reactions were carried out in the absence of AfmE1 and run in parallel.

CZE and TLC experiments were carried out in order to define the AfmE1 mode of action. Regarding the cleavage of cello-oligosaccharides, the enzyme acts on oligosaccharides containing five or more glucose units (Fig 3). However, the hydrolysis efficiency was higher for cellohexaose (C6) than for cellopentaose (C5), with rates of 16.7 nmol.min^-1^.mg^-1^ and 22.7 nmol.min^-1^.mg^-1^ for C5 and C6, respectively. Under the same experimental conditions, all C6 oligosaccharides were cleaved after 2 h, while a significant amount of C5 remained intact even after 4 h of reaction (Fig 3). This difference towards C5 and C6 suggests that the active site might accommodate productively six glucosyl residues.

The cleavage pattern showed to be similar to that of canonical endo-β-1,4-glucanases, yielding mainly cellotriose, cellotetraose and cellobiose from C6 hydrolysis. Using C5 as substrate, the main products of hydrolysis were cellobiose and cellotriose. The enzyme was not active on cellobiose, cellotriose or cellotetraose (Fig 3). The cleavage pattern for the substrates β-glucan and CMC in the presence of AfmE1 was also investigated (Fig 3F and 3G). The main products resulting from the hydrolysis of both substrates were C4, C3 and C2, confirming that the enzyme is unable to hydrolyze cello-oligosaccharides shorter than 5 units. Furthermore, in the case of β-glucan, there was an accumulation of oligosaccharides higher than C6. This fact may be attributed to the presence of β-1,3 glycosidic bonds in these oligosaccharides, which may interfere with enzymatic cleavage. Our data also demonstrate that AfmE1 presents an endo mode of action since substrate degradation produced oligosaccharides of various lengths, indicating the enzymatic cleavage of internal glycosidic bonds randomly along the polysaccharide chain.

The comparative analysis of substrate degradation with other endoglucanases from GH8 family unveiled some differences and similarities between these enzymes. As an example, the enzyme CelC from *Clostridium cellulolyticum* also hydrolyzes β-glucan and CMC more efficiently than lichenan. Differently from AfmE1, this enzyme can hydrolyze cellotetraose and cellotriose [37]. The enzymes Egl-257 and Endo-K from *Bacillus*, on the other hand, degrade lichenan as efficiently as–or more efficiently than–CMC. Regarding the cleavage of oligosaccharides, both enzymes act on cellohexaose and cellopentaose like AfmE1, although Endo-K also slightly degrades cellotetraose [38, 41]. These differences indicate that, despite belonging to the same family, each enzyme presents specific features concerning substrate recognition and processing.

### Thermal stability and hydrodynamic behavior of AfmE1

**Fig 4:**
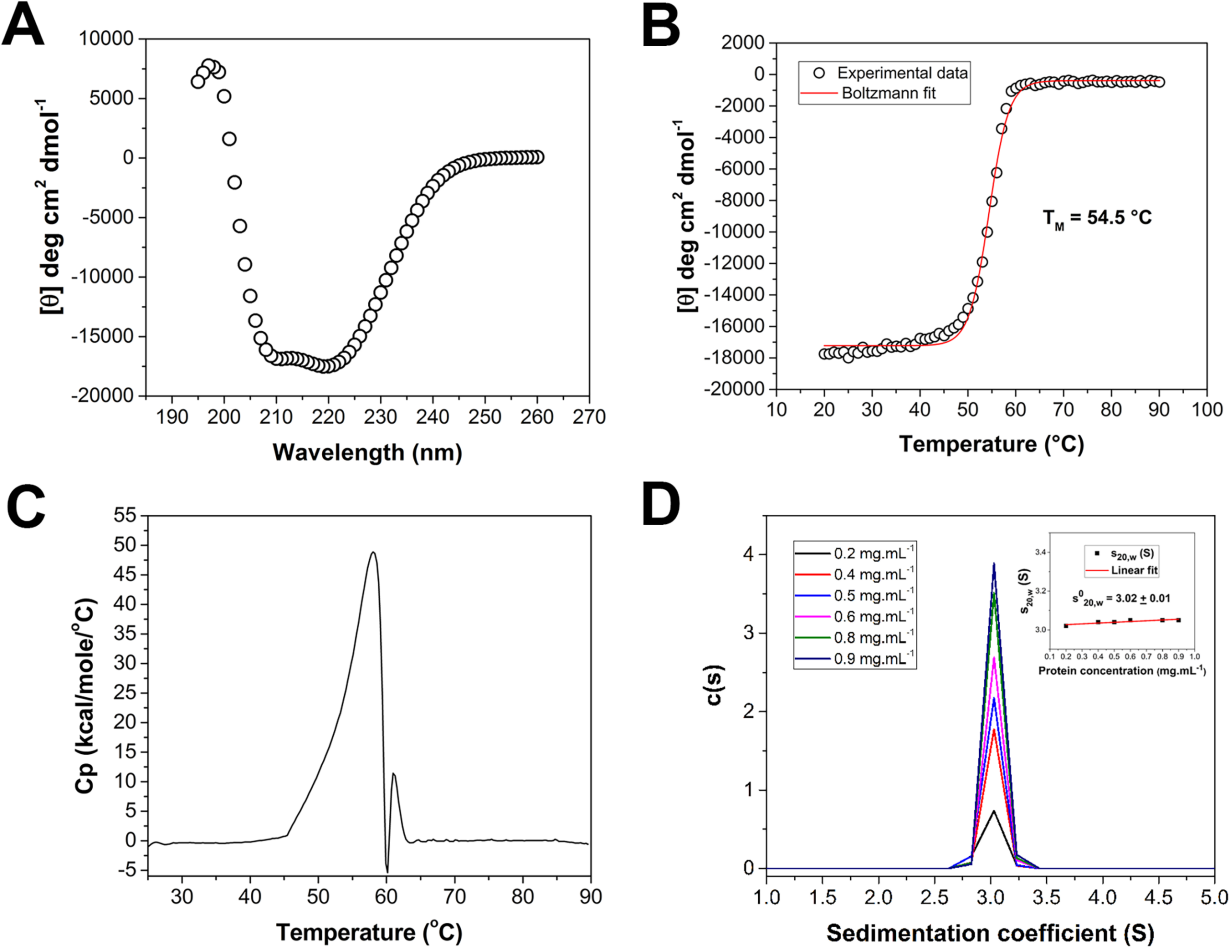
Biophysical characterization. (A) Circular dichroism spectrum of AfmE1 indicating that the recombinant protein was produced and purified in a folded conformation. CD (B) and DSC (C) thermal unfolding curves showed similar melting temperatures around 55°C. The second peak in the DSC curve corresponds to protein aggregation after denaturation. (D) AUC analysis of AfmE1 at different concentrations confirms that the protein is monomeric in solution with a molecular weight of approximately 39 kDa.

Biophysical studies were performed to verify protein thermal stability and hydrodynamic behavior. CD analysis of AfmE1 showed a far-UV spectrum characteristic of an α-helical rich protein with two minima at 208 and 222 nm, as expected for GH8 family members (Fig 4A). CD thermal unfolding experiments indicated a melting temperature (*T*_M_) around 55°C (Fig 4B). A similar melting temperature was also obtained by DSC and in both cases enzyme denaturation showed to be an irreversible process defined by a two-state unfolding model (Fig 4C). These results are in accordance with our functional results showing a fast activity loss at temperatures higher than 50°C (Fig 2C).

To investigate the hydrodynamic behavior and oligomeric state of AfmE1, sedimentation velocity AUC experiments were carried out. From the distribution curves at different protein concentrations, it was calculated an *s*^0^_20,w_ of 3.02±0.01 S and a molecular mass of 39±1 kDa (*f*/*f*_0_ = 1.36±0.1) (Fig 4D). These values are compatible to the monomeric state of AfmE1, which is similar to that observed for other structurally characterized GH8 members, indicating that oligomerization is not a feature in this family.

### AfmE1 shares high structural similarity to endoglucanases belonging to the Bcs complex

**Fig 5:**
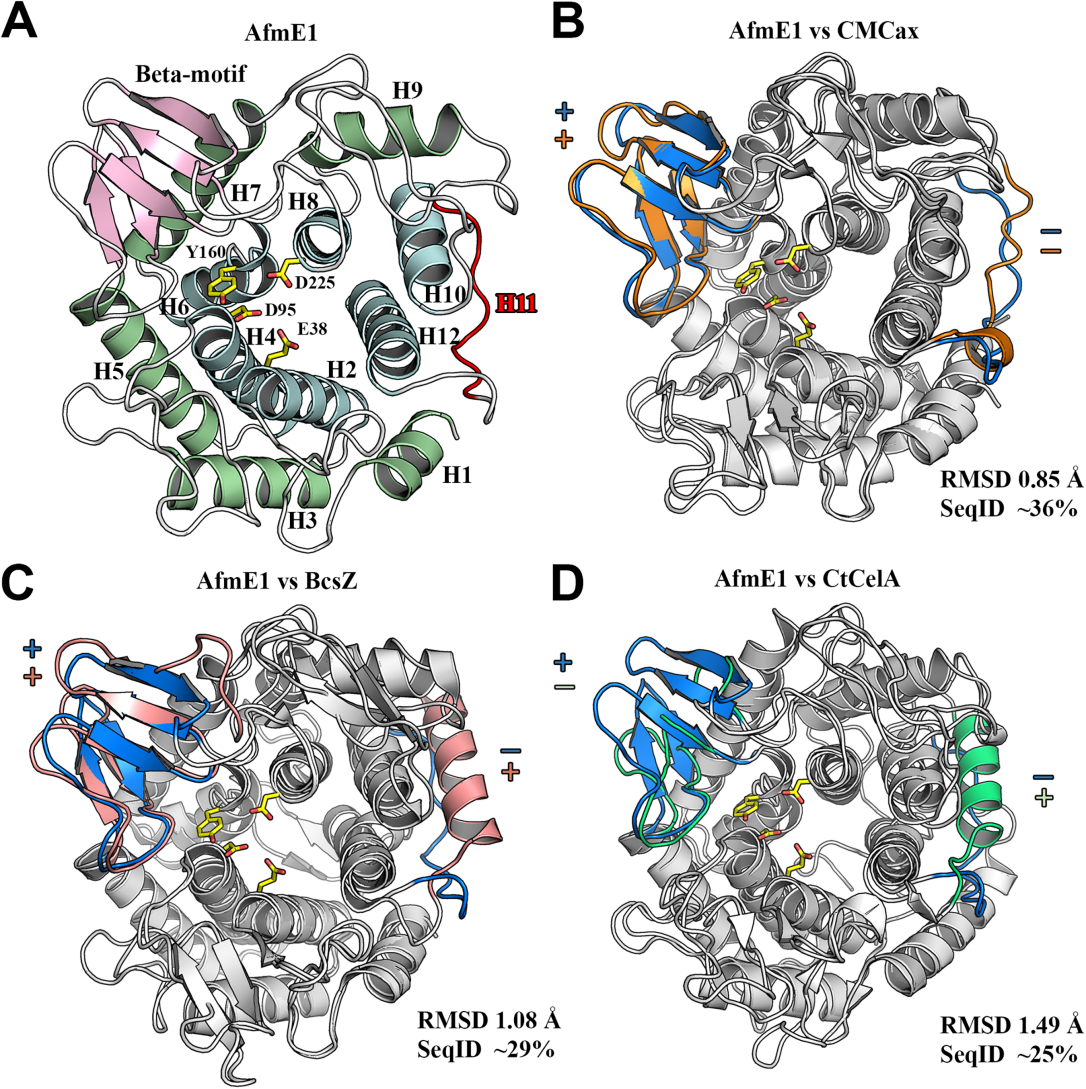
The crystallographic structure of AfmE1. (A) Cartoon representation of the AfmE1 (α/α)_6_ barrel fold with the inner and outer helices colored in light-blue and light-green, respectively. The β-motif is shown in pink and the region of the lacking helix-α11 in red. The residues critical for catalysis are depicted as sticks with carbon atoms in yellow. Superposition of AfmE1 structure with those of *K*. *xylinus* CMCax (PDB code 1WZZ) (B), *E*. *coli* BcsZ (PDB code 3QXQ) (C) and *C*. *thermocellum* CelA (PDB code 1CEM) (D). The presence (+) or absence (-) of the β-motif and helix-α11 is indicated and highlighted in blue, orange, pink and green in the structures of AfmE1, CMCax, BcsZ and CelA, respectively. The r.m.s.d. and sequence identity values for each structural alignment are indicated.

The crystallographic structure of AfmE1 was determined at 2.39 Å resolution with *R*/*R*_free_ of 0.20/0.24 and excellent overall stereochemistry (Table 1). AfmE1 folds into an (α/α)_6_ barrel that consists of six pairs of antiparallel α-helices forming an inner and outer ring (Fig 5A). This molecular architecture is common for inverting glycosidases and has also been described for enzymes belonging to families GH9, GH15, GH37, GH48, GH63, GH65, GH94 and GH125 (CAZy Database; [43]). In the case of AfmE1, however, one of the short external helices (α11, by homology with other enzymes) is missing (Fig 5A). Furthermore, the helix α1 is shorter than observed in its homologues, but this conformational change seems to be associated to crystal packing effects since the unfolded segment is embedded in the symmetry molecule performing cation-π interactions. AfmE1 structure also includes a β-motif that, in conjunction with the loops connecting the α-helices and β-strands, delineate the substrate-binding cleft. This groove constitutes the most conserved region of the molecule and the catalytic residues Glu38 (proton donor) and Asp225 (proton acceptor/general base) are located at its center. The conserved residues Asp95 and Tyr160 also lay in this region and are important for substrate breakdown, establishing hydrogen bonds that are crucial for the correct positioning of the glucosyl residue in the subsite -1 and the nucleophilic water molecule, respectively [44, 45] (Fig 5A).

Adachi et al [46] showed that the proton acceptor position can vary among members of the GH8 family and proposed its division into 3 subfamilies, based on a phylogenetic analysis. According to this classification, AfmE1 belongs to group GH8a, as the catalytic aspartate, Asp225, occupies the same position of Asp278 in the enzyme CelA from *C*. *thermocellum*. Molecular dynamics data support the role of this aspartate as the proton acceptor within this group and suggest the existence of a general mechanism of catalysis that could be applied to all GH8 members [47].

Despite sharing low amino acid sequence identity with other structurally characterized GH8 enzymes, AfmE1 showed high structural similarity to endoglucanases from the family GH8, especially those that integrate Bcs complexes (Fig 5). The structure superposition of AfmE1 on CMCax from *K*. *xylinus* and BcsZ from *E*. *coli* resulted in an overall RMSD of 0.85 Å and 1.08 Å, respectively, while the structural alignment with CelA from *C*. *thermocellum*, a non-Bcs cellulase, exhibited a higher average displacement (RMSD = 1.49 Å). The main differences between these structures were found in the regions of the β-motif and helix α-11. AfmE1 and CMCax structures are very similar as both proteins exhibited the presence of the β-motif and the absence of helix α-11 (Fig 5B). BcsZ and CelA differed from AfmE1 due to the presence of helix α-11 and CelA also differed from the other enzymes due to the absence of the β-motif (Fig 5C and 5D). This high structural similarity of AfmE1 to endoglucanases of the Bcs complex suggests that these enzymes may share similar functional roles.

### AfmE1 substrate-binding cleft comprises 6 subsites with an atypical configuration at position +3

GH8 enzymes cleave the glycosidic bond via a single displacement mechanism with inversion of the anomeric configuration. The current understanding about this reaction mechanism including the structural determinants for substrate interaction are mainly based on studies of the cellulase CelA from *C*. *thermocellum*, with crystal structures available for its free, substrate-, and product-bound forms [44, 48]. Furthermore, structural studies of the wild-type and mutated forms of the xylanase pXyl from the Antarctic bacterium *P*. *haloplanktis* [45, 49, 50] also contributed to establish the role of several active-site residues that are conserved in the GH8 family. These studies have shown that while the enzyme undergoes small structural changes upon carbohydrate binding, the active site conformation forces the oligosaccharide chain to kink, leading to a distortion of the glucosyl residue in the subsite -1 from the ground-state ^4^C_1_ to a ^2,5^B conformation in the case of cellulose CelA. For pXyl, sugar distortion was not so drastic but affected the xylosyl residues at two consecutive subsites (-1 and -2).

**Fig 6:**
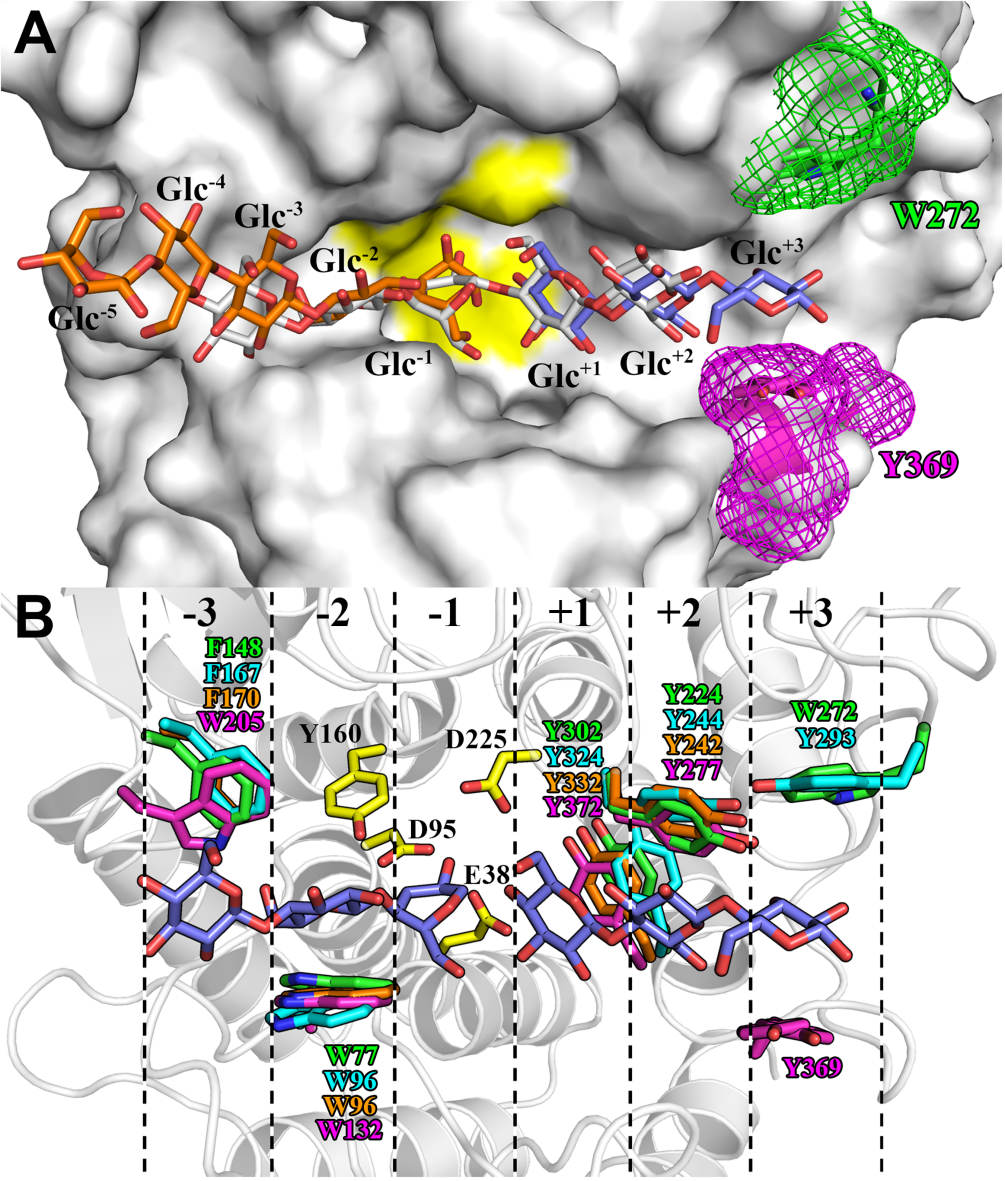
AfmE1 substrate-binding cleft. (A) Molecular surface of AfmE1 with its stacking residue Trp272 in subsite +3 represented as sticks with carbon atoms in green. The stacking residue Tyr369 in the corresponding region of CelA from *C*. *thermocellum* is similarly represented in magenta to evidence the differences in the subsite +3 configuration of these enzymes. The region containing the catalytic residues is highlighted in yellow. The substrate molecules, represented as deduced from the complex of CelA with cellopentaose (white) and cellotriose (blue) (PDB code 1KWF), as well as of BcsZ with cellopentaose (orange) (PDB code 3QXQ), are shown as sticks to indicate the position of the subsites. (B) AfmE1 substrate-binding cleft highlighting the catalytic (yellow) and the glucosyl-stacking residues (green) in its six subsites (dashed lines). The corresponding stacking residues of the proteins CMCax, BcsZ and CelA are shown in cyan, orange and magenta, respectively. The position of the glucosyl residues (blue) occupying the six subsites was predicted from the complex CelA-substrate [44].

Protein-carbohydrate stacking interactions play an essential role in the productive binding and stabilization of sugar chains. Similarly to the enzyme CelA from *C*. *thermocellum*, AfmE1 cleaves oligosaccharides containing 5 or more glycosyl residues, indicating the presence of at least 5 critical substrate-binding subsites for catalysis [44]. In the structure of CelA-cellopentaose complex, the substrate was modelled occupying the positions -3 to +2, whereas a cellotriose molecule, mimicking the leaving group, was modelled bound to subsites +1 to +3 (Fig 6A). The structure of *E*. *coli* BcsZ was also solved in the presence of cellopentaose, although in this case the carbohydrate is atypically bound to the enzyme, interacting exclusively with residues on the nonreducing end of the catalytic groove (Fig 6A). Mazur & Zimmer [10] speculated that this interaction could reflect a post-hydrolysis state in which the leaving group has already dissociated from the enzyme-substrate complex.

Structural superposition of the substrate-binding cleft of AfmE1 to that of other GH8 endoglucanases showed that the residues involved in protein-sugar stacking interactions are highly conserved at positions -2, +1 and +2 (Fig 6B). The subsite -3 of CelA differed from the other enzymes due to the replacement of the phenylalanine residue by a tryptophan. However, the most notable differences were found in stacking residues at the subsite +3, which varied both in amino acid composition and location. Interestingly, although an aromatic residue corresponding to Tyr369 of CelA is missing in the AfmE1 structure, the residue Trp272 on the opposite side of the catalytic cleft is properly positioned to establish a stacking interaction with the glucosyl residue in the subsite +3 (Fig 6A). Yasutake et al. [24] reported the absence of this subsite in the structure of CMCax. However, this protein has a tyrosine (Tyr293) positioned in the same way as Trp272 of AfmE1. Only in the protein BcsZ from *E*. *coli* the subsite +3 seems to be really missing as there is no aromatic residue in the corresponding region of the molecule (Fig 6B).

The fact that this subsite is not conserved among GH8 endoglucanases suggests that it is not critical to the productive binding of the substrate. However, its presence contributes to some extent in this process, since AfmE1 presents a higher hydrolytic rate against cellohexaose than cellopentaose. In addition, the alternative cleavage pattern of cellohexaose, which produced both C3 + C3 or C2 + C4, indicates that the subsite +3 is used only in part of the cleavage reactions. Interestingly, in the case of the enzyme BcsZ from *E*. *coli*, which lacks this subsite, the catalytic activity against cellulose was not detected in liquid assays but only when the substrate was embedded in agar plates [10]. According to the authors, the heating process during agar plate preparation could partially dissociate the cellulose microfibrils suggesting that this enzyme hydrolyzes cellulose in a loosened configuration. Therefore, it is conceivable that the variability in the cellulose binding site could influence the recognition and processing of molecules with different arrangements. In this sense, the presence of a gene coding for an endoglucanase sharing a high similarity to BcsZ (72% identity and 83% similarity) close to AfmE1 sequence, but in a distinct Bcs operon in the genome of *R*. *ornithinolytica* (Fig 1), may suggest that these enzymes integrate different Bcs complexes and are possibly involved in the synthesis of structurally different cellulose chains. According to Römling & Galperin [1], the structural divergence of Bcs enzymes, in conjunction with the great variety of cellulose synthase operons in bacteria, might reflect the diversity of cellulose products and their different arrangements and crystallinities. Although the process of bacterial cellulose biosynthesis has not been completely elucidated, studies involving microorganisms from genus *Komagataeibacter* have demonstrated the production of bacterial cellulose with distinct characteristics (crystallinity, thickness, tensile strength, among others), depending on growth conditions such as the carbon and nitrogen sources, media additives, etc. [51, 52, 53]. This fact suggests that different Bcs operons may be activated upon changes in environmental factors, producing cellulose fibrils with different structural properties. Further studies are necessary to elucidate the molecular mechanisms involved in the biosynthesis of distinct cellulose products, which might impact on many relevant areas such as bacterium-host interaction, biofilm formation and design of cellulosic materials for biomedical and biotechnological applications.

## Supporting information

S1 Fig Kinetic curves for the reactions of AfmE1 against β-glucan, CMC 4M and lichenan. The Michaelis-Menten parameters were determined from the saturation curve obtained using different β-glucan concentrations. In the kinetic analyses using CMC or lichenan as substrate, saturation was not achieved as the high viscosity interfered with the determination of activity at higher substrate concentrations.
(PDF)
