# Supplementary material for: Structure and function of a novel GH8 endoglucanase from the bacterial cellulose synthase complex of Raoultella ornithinolytica
Source: PLoS One. 2017 Apr 27;12(4):e0176550. doi: 10.1371/journal.pone.0176550 (PMC5407803; doi:10.1371/journal.pone.0176550)
Supplement: S1 Fig — The Michaelis-Menten parameters were determined from the saturation curve obtained using different β-glucan concentrations. In the kinetic analyses using CMC or lichenan as substrate, saturation was not achieved as the high viscosity interfered with the determination of activity at higher substrate concentrations. (PDF) [file pone.0176550.s001.pdf]

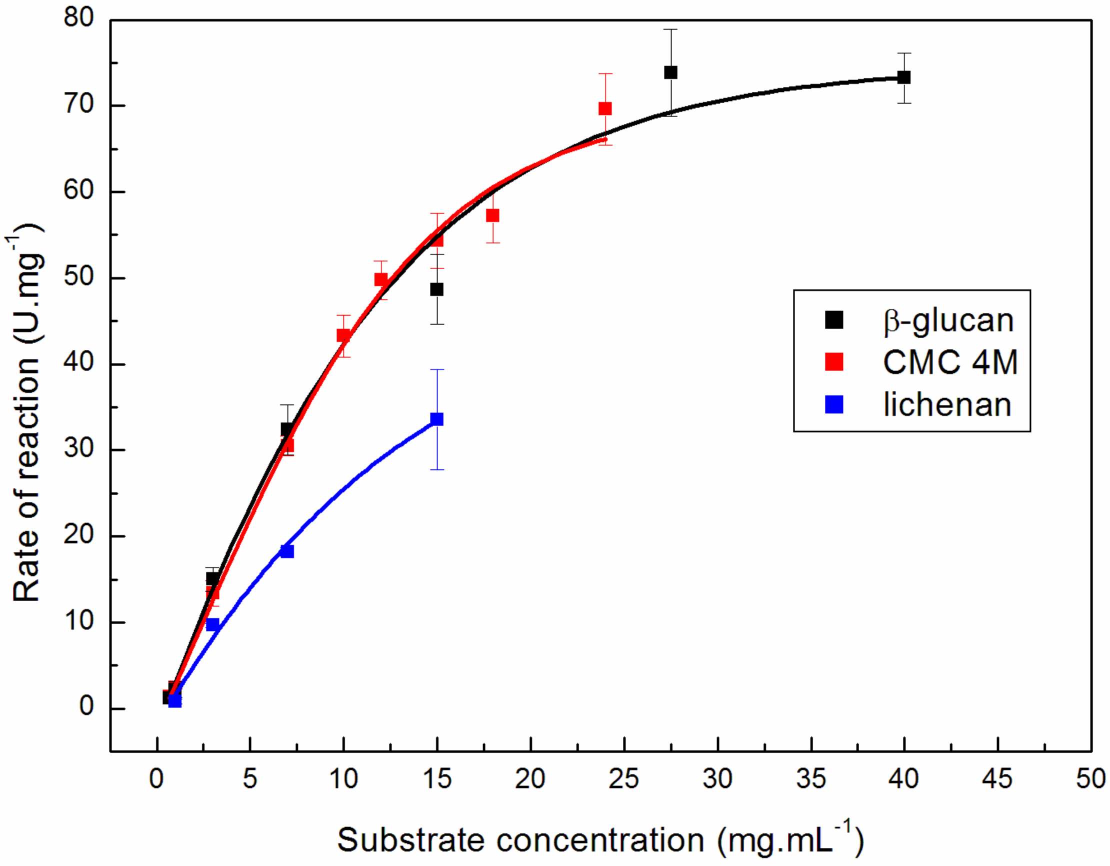

S1 Fig: Kinetic curves for the reactions of AfmE1 against β-glucan, CMC 4M and lichenan. The Michaelis-Menten parameters were determined from the saturation curve obtained using different β-glucan concentrations. In the kinetic analyses using CMC or lichenan as substrate, saturation was not achieved as the high viscosity interfered with the determination of activity at higher substrate concentrations.
